# Duodenal metastasis from breast carcinoma presenting with refractory nausea and vomiting: a case report

**DOI:** 10.1093/jscr/rjag210

**Published:** 2026-03-29

**Authors:** Su Su (Shannon) Naing

**Affiliations:** Department of General Surgery, The Hervey Bay Hospital, Hervey Bay, Cnr Nissen Street, &, 141/169 Urraween Rd, Pialba QLD 4655, Australia

**Keywords:** breast carcinoma, duodenal metastasis, immunohistochemistry, GATA3, case report

## Abstract

Breast carcinoma rarely metastasises to the gastrointestinal tract, and duodenal involvement is particularly uncommon. We describe a patient with a complex history of recurrent invasive ductal breast carcinoma who presented with persistent nausea and vomiting refractory to conventional therapy. Endoscopic evaluation revealed mucosal nodularity in the second portion of the duodenum. Biopsies confirmed metastatic breast carcinoma immunohistochemically positive for GATA3 and mammaglobin, and negative for CK20, indicating breast origin. This case highlights the importance of considering gastrointestinal metastasis in patients with prior breast cancer presenting with gastrointestinal symptoms. Early endoscopic assessment and immunohistochemical confirmation were key to diagnosis. Clinicians should maintain a high index of suspicion for atypical metastatic patterns in patients with advanced breast carcinoma.

## Introduction

Breast carcinoma most commonly metastasises to bone, lung, liver, and brain [1]. Gastrointestinal (GI) involvement is rare, with reported incidence ranging from 4% to 18% in autopsy studies, though symptomatic GI metastases are uncommon [1, 2]. The stomach is the most frequently affected site, while duodenal metastasis is particularly unusual [2, 3]. Such presentations may mimic primary GI malignancy, creating diagnostic challenges [3, 4].

This case demonstrates diagnostic challenges in a patient with extensive oncological history presenting with non-specific GI symptoms, and highlights tumor receptor discordance over disease progression.

## Case report

A woman in her fifties was referred by her oncologist with refractory nausea and vomiting limiting her oral intake. She had complex breast cancer history; she was first diagnosed with right breast human epidermal growth factor receptor 2 (HER2) positive cancer which was treated with neoadjuvant chemotherapy, lumpectomy, and axillary lymph node dissection followed by radiotherapy in 2007. In 2011, she developed bilateral breast cancer with different hormone receptor status: left breast invasive ductal carcinoma (IDC) estrogen receptor (ER) positive, progesterone receptor (PR) positive, HER2 negative, and right breast IDC with HER2 positive. She then underwent bilateral mastectomies and left axillary lymph node dissection followed by adjuvant chemotherapy and immunotherapy. In 2015, she was diagnosed with stage 4 breast cancer with metastasis in mediastinum and inguinal lymph nodes. She was then treated again with chemotherapy and immunotherapy followed by maintenance trastuzumab and pertuzumab as she achieved complete radiological response. She had a background of autoimmune disease which includes Raynaud’s syndrome and respiratory problem.

**Figure 1:**
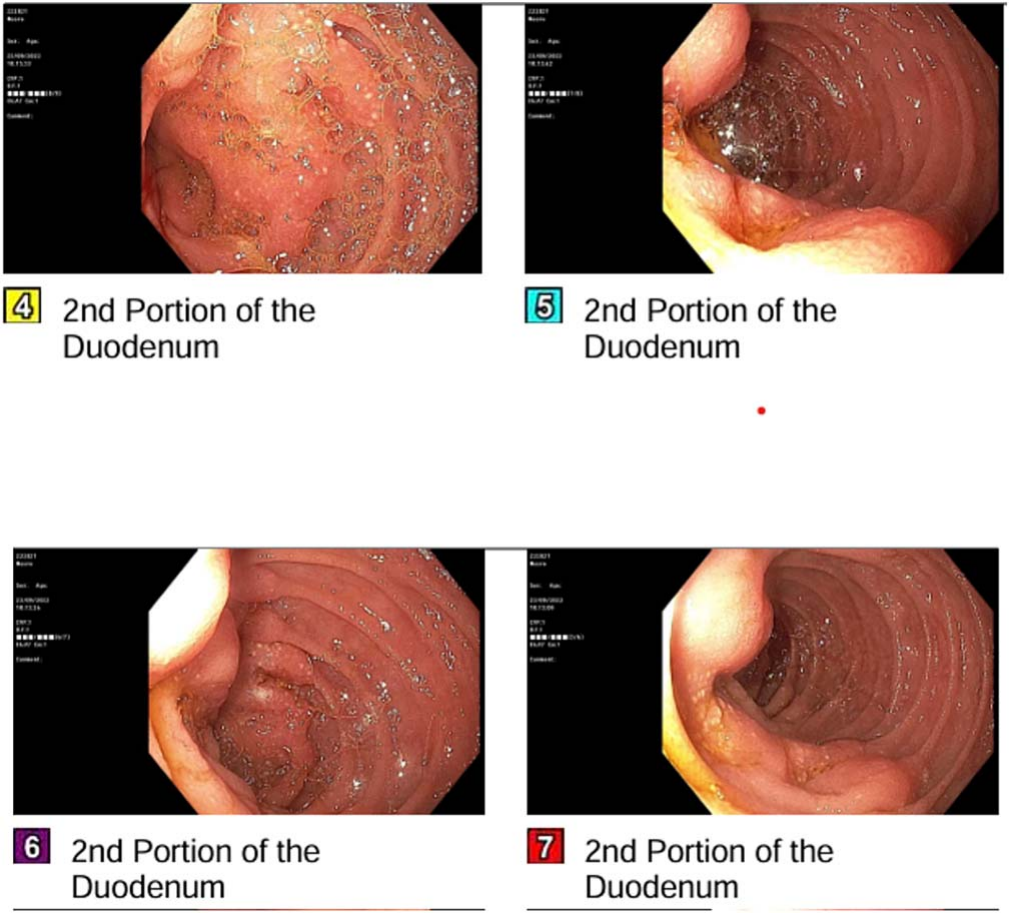
Endoscopic appearance of duodenal metastasis. Segmental mucosal nodularity and altered texture observed in the second portion of the duodenum, with scattered white speckled lesions.

**Figure 2:**
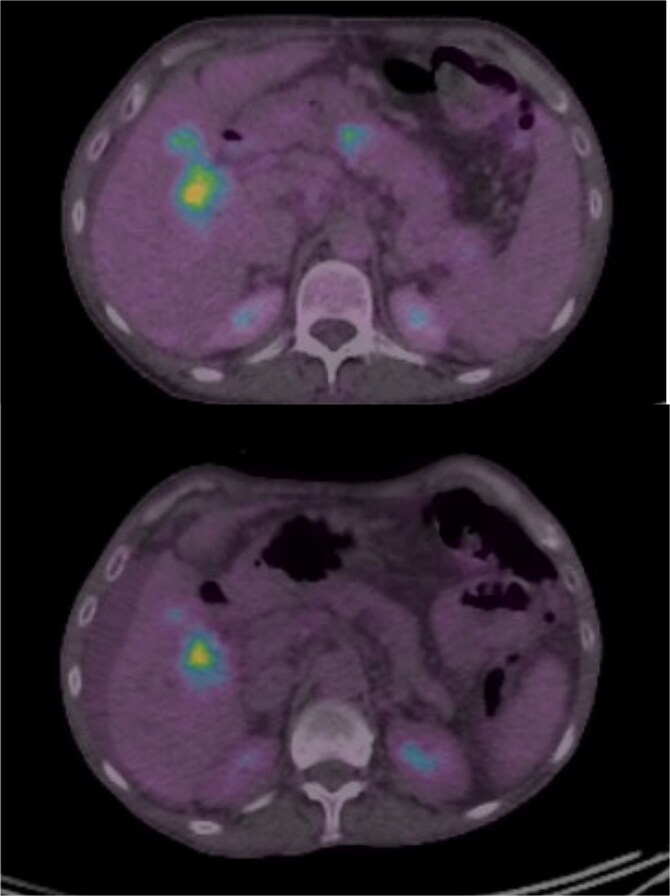
PET-CT scan showing systemic metastatic disease. Metabolic activity in mesenteric, retroperitoneal, and hepatic regions confirming progressive systemic disease.

She developed persistent nausea and vomiting unresponsive to antiemetic therapy; therefore, she was referred to the surgical department for endoscopy and colonoscopy. Endoscopy revealed segmental mucosal changes in the second portion of the duodenum (Fig. 1). Biopsy specimens demonstrated metastatic carcinoma. Immunohistochemistry was positive for GATA3, mammaglobin, and CK7, and negative for CK20 and ER consistent with metastatic breast origin. A positron emission tomography (PET) scan (Fig. 2) revealed widespread nodal and hepatic metastatic disease. The patient subsequently died due to progressive disease.

## Discussion

Breast cancer metastasis to the GI tract is a rare clinical entity, particularly when involving the duodenum. While autopsy series suggest GI metastases may occur in up to 16%–16.4% of breast cancer fatalities, the clinical detection rate is significantly lower, estimated at ~ 0.3%–1% [1, 2]. Among these, the stomach and colon are the most common sites, making solitary duodenal metastasis an exceptionally infrequent presentation [3]. This case illustrates the diagnostic complexity of such metastases, particularly in patients with a remote and complex history of breast cancer.

The clinical presentation of duodenal metastasis is often non-specific, mimicking primary GI malignancies or benign conditions. As seen in this patient, symptoms typically include refractory nausea, vomiting, epigastric pain, or bleeding [2, 4]. Endoscopic findings can be variable, ranging from mucosal thickening and extrinsic compression to ulceration or discrete nodules [4]. Because these findings are indistinguishable from primary duodenal adenocarcinomas on gross inspection, histopathological evaluation with immunohistochemistry (IHC) is essential for definitive diagnosis.

Differentiating metastatic breast cancer from primary GI malignancies relies heavily on specific IHC markers. Metastatic breast carcinoma typically exhibits a cytokeratin 7 (CK7) positive and cytokeratin 20 (CK20) negative immunophenotype, whereas primary intestinal adenocarcinomas are generally CK7 negative and CK20 positive [5]. In this case, the tumor was CK7 positive and CK20 negative, strongly supporting a breast origin. Furthermore, the expression of GATA3 and mammaglobin provided high specificity; GATA3 is a sensitive marker for breast and urothelial carcinomas, while mammaglobin is highly specific for breast tissue [5, 6]. The combination of these markers confirmed the diagnosis in the absence of a distinct primary breast lesion at the time of presentation.

A critical aspect of this case is the discordance in hormone receptor status between the primary tumors and the metastatic site. The patient had a history of heterogenous disease, including HER2-positive and ER-positive primaries. The duodenal metastasis, however, was ER-negative. Receptor conversion (discordance) is a documented phenomenon in metastatic breast cancer, with studies reporting a change in ER, PR, or HER2 status in 14%–30% of metastatic lesions compared to the primary tumor [7, 8]. This heterogeneity highlights the importance of biopsy and re-evaluation of metastatic sites, as changes in receptor status can fundamentally alter therapeutic options and prognosis [8]. In this patient, the negative ER status of the metastasis may represent either a clonal selection of the ER-negative clone (from her right-sided disease) or a true phenotypic drift of the ER-positive disease.

Historically, invasive lobular carcinoma (ILC) has a greater propensity for GI metastasis compared to IDC, with some series suggesting ILC accounts for the majority of GI metastases despite representing a minority of primary breast cancers [3, 4]. However, as demonstrated in this patient with a history of IDC, ductal carcinoma can also metastasize to the small bowel.

In conclusion, duodenal metastasis from breast cancer should be considered in the differential diagnosis for any patient with a history of breast malignancy presenting with refractory upper GI symptoms. This case underscores the necessity of endoscopic biopsy with a comprehensive IHC panel—including GATA3, mammaglobin, CK7, and CK20—and determining current receptor status to guide appropriate systemic management.

## Conclusion

Duodenal metastasis from breast carcinoma is rare but should be considered in patients presenting with refractory GI symptoms and a prior history of breast malignancy. Endoscopic biopsy with immunohistochemical analysis is crucial for diagnosis. Awareness of receptor discordance during disease progression is important for guiding management [5, 6].
